# Cardiac Contractility Modulation (CCM) Therapy in Contemporary Heart Failure Care: Mechanisms, Evidence, Patient Selection, and Emerging Directions

**DOI:** 10.3390/jcm15041460

**Published:** 2026-02-13

**Authors:** Dong-Hyeok Kim, Yeji Kim, Jungmin Kang, Junbeom Park

**Affiliations:** 1 Division of Cardiology, Ewha Womans University Seoul Hospital, Seoul 07804, Republic of Korea; tomas9912@naver.com; 2 Division of Cardiology, Ewha Womans University Mokdong Hospital, Seoul 07985, Republic of Korea; angelak6506@gmail.com (J.K.); newriser@naver.com (J.P.)

**Keywords:** cardiac contractility modulation (CCM), heart failure, device therapy, quality of life, exercise capacity, remodeling

## Abstract

Cardiac contractility modulation (CCM) is a bioelectronic therapy that delivers precisely timed electrical signals during ventricular refractoriness to modulate myocardial contractility without triggering depolarization. Unlike pacing-based therapies, CCM does not initiate a new depolarization but instead modulates intracellular signaling pathways to enhance myocardial contractility without increasing myocardial oxygen consumption. CCM therefore represents a myocardial conditioning strategy distinct from cardiac resynchronization therapy, conduction system pacing, or neuromodulation. Experimental and translational studies demonstrate that repeated CCM delivery induces sustained myocardial adaptations, including improvements in excitation–contraction coupling, molecular signaling pathways, and structural remodeling that extend beyond transient hemodynamic effects. Across clinical investigations, CCM has been associated with meaningful improvements in exercise tolerance, health-related quality of life, and functional status in carefully selected populations. Observational data further suggest a potential reduction in heart failure-related hospitalizations when therapy is applied within evidence-aligned indications. Recent technological developments—including simplified ventricular lead configurations, rechargeable compact generators, and integrated CCM–defibrillator platforms—have reduced procedural complexity and may broaden clinical applicability, particularly in patients with concomitant implantable cardioverter–defibrillator indications. This review synthesizes mechanistic insights, clinical evidence, patient selection principles, and practical considerations to define the evolving role of CCM within contemporary heart failure care pathways.

## 1. Introduction

Despite the expansion of guideline-directed medical therapy (GDMT), many patients with heart failure with reduced ejection fraction (HFrEF) continue to experience HF symptoms (e.g., dyspnea and shortness of breath) and recurrent decompensation requiring hospitalization [1,2]. While device-based strategies such as cardiac resynchronization therapy (CRT) confer substantial benefit in appropriately selected populations, a considerable proportion of symptomatic patients do not fulfill electrical criteria for CRT—most commonly because of narrow QRS duration or non-left bundle branch block conduction patterns [2,3]. Consequently, a clinically relevant subgroup remains in whom therapeutic options beyond pharmacological optimization and established resynchronization strategies are limited. Throughout this manuscript, heart failure phenotypes are defined using contemporary guideline terminology: heart failure with reduced ejection fraction (HFrEF) refers to a left ventricular ejection fraction (LVEF) ≤ 40%, whereas heart failure with mildly reduced ejection fraction (HFmrEF) refers to an LVEF of 41–49%.

Cardiac contractility modulation (CCM) was developed to address this unmet need by applying precisely timed electrical signals to the ventricular myocardium during refractoriness, thereby influencing myocardial performance without generating an additional depolarization [4,5]. In contrast to therapies that primarily target electrical synchrony, CCM is designed to influence myocardial contractile behavior at the cellular and molecular levels, offering a complementary strategy for patients who are not candidates for CRT. Cardiac contractility modulation (CCM) is a bioelectronic therapy that delivers non-excitatory electrical signals to the ventricular myocardium during the absolute refractory period. Unlike pacing-based therapies, CCM does not initiate a new depolarization but instead modulates intracellular signaling pathways to enhance myocardial contractility without increasing myocardial oxygen consumption. CCM therefore represents a myocardial conditioning strategy distinct from cardiac resynchronization therapy, conduction system pacing, or neuromodulation.

**Figure 1:**
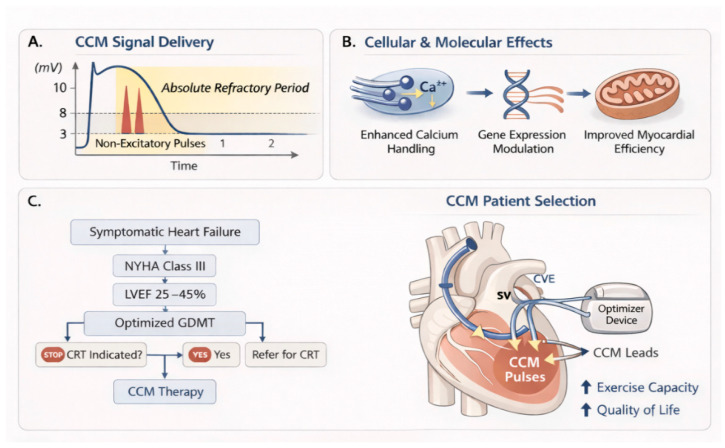
Conceptual mechanism and clinical positioning of cardiac contractility modulation therapy in contemporary heart failure management (Courtesy of AI, ChatGPT 5.2). This figure illustrates the fundamental principles of cardiac contractility modulation (CCM) therapy. CCM delivers non-excitatory electrical signals during the absolute refractory period, leading to multi-level myocardial effects as cellular calcium handling, gene expression modulation, myocardial energetics, and macroscopic reverse remodeling, without altering intrinsic rhythm. Experimental and translational studies suggest that CCM exerts multi-level myocardial effects. At the cellular level, CCM enhances calcium handling through modulation of sarcoplasmic reticulum calcium cycling and calcium-sensitive signaling pathways, including CaMKII-related mechanisms. At the molecular level, CCM has been associated with favorable changes in gene expression profiles related to excitation–contraction coupling and myocardial energetics. At the tissue level, these effects may translate into reverse remodeling and improved contractile efficiency. At the clinical level, CCM fills an important therapeutic gap for patients with symptomatic heart failure who are not candidates for CRT, offering improvements in exercise capacity and quality of life through a distinct bioelectronic mechanism. (**A**): Timing of CCM signal delivery during the absolute refractory period of the ventricular action potential, illustrating non-excitatory high-voltage impulses that do not trigger a new depolarization. (**B**): Cellular and molecular effects of CCM, including enhanced calcium handling, normalization of gene expression, and improved myocardial efficiency. (**C**): Clinical positioning algorithm highlighting symptomatic heart failure patients (NYHA class III) with LVEF 25–45%, spanning the upper HFrEF and lower HFmrEF spectrum, who remain symptomatic despite GDMT and do not meet criteria for CRT, representing the primary CCM target population. This figure is original artwork created specifically for this manuscript using AI-assisted illustration (ChatGPT 5.2, developed by OpenAI, Inc., San Francisco, CA, USA). It is not adapted from any previously published material, and no external permissions are required.

Growing interest in CCM reflects recognition that its target population overlaps only partially with other contemporary device-based interventions, including CRT, conduction system pacing, and advanced heart failure therapies [4,6]. This distinct positioning—situated between pharmacologic optimization and resynchronization-based approaches—forms the conceptual framework illustrated in Figure 1 and has prompted renewed evaluation of CCM as a component of individualized, mechanism-oriented heart failure management.

## 2. What Is CCM? Signal Characteristics and System Components

Cardiac contractility modulation is delivered through non-excitatory electrical signals applied during the absolute refractory period of the ventricular action potential, permitting modulation of myocardial performance without initiating a new depolarization. This timing strategy permits signal delivery without initiating myocardial depolarization and is achieved using an implantable pulse generator connected to dedicated ventricular septal leads [4,7]. By confining stimulation to myocardial refractoriness, CCM avoids the generation of paced ventricular complexes while influencing downstream processes related to myocardial contractile behavior. CCM does not function as a pacing modality. Instead, it is conceptualized as a myocardial conditioning therapy that modulates intracellular signaling pathways involved in cardiac contraction and myocardial adaptation [4,5].

Contemporary CCM platforms have evolved to incorporate programmable algorithms that support individualized optimization and longitudinal follow-up. Commercially available systems include the OPTIMIZER Smart device family, with newer iterations emphasizing reduced generator size, rechargeable power sources, and enhanced programmer interfaces to facilitate chronic therapy delivery in routine clinical practice [8,9,10,11].

In the United States, regulatory approval has focused on patients with symptomatic heart failure despite guideline-directed medical therapy who do not meet established criteria for cardiac resynchronization therapy. The specified left ventricular ejection fraction (LVEF) range reflects responder-enrichment strategies derived from pivotal clinical trials, aiming to identify individuals most likely to derive sustained clinical benefit from CCM therapy [7,9,10].

## 3. Mechanisms of Action: From Acute Effects to Reverse Remodeling

Building on this signal delivery paradigm, CCM induces downstream effects on calcium handling, gene expression, and myocardial energetics that extend beyond acute hemodynamic modulation (Table 1).

Experimental investigations in animal models of heart failure provided early evidence that repetitive CCM therapy can produce durable improvements in ventricular performance and geometry, supporting a biological paradigm that extends beyond transient contractile augmentation [5].

Although CCM signals are delivered during the absolute refractory period and do not elicit a new action potential, subthreshold electrical stimulation can nonetheless influence the excitation–contraction coupling apparatus. Experimental studies suggest that the applied electrical field may directly modulate intracellular electrochemical processes, particularly calcium handling within the sarcoplasmic reticulum, without requiring membrane depolarization. Such electrochemical effects may alter calcium transient amplitude and kinetics, thereby engaging calcium-dependent signaling cascades that persist beyond the immediate stimulus.

In addition to electrochemical mechanisms, a contributory role for mechano-electrical coupling has been proposed. Localized myocardial deformation induced by septal stimulation during systole may activate stretch-sensitive pathways, further influencing intracellular signaling and gene expression. Importantly, these electrochemical and mechano-electrical effects are not mutually exclusive and likely operate in concert, providing a plausible explanation for how CCM can modify myocardial contractile behavior and promote longer-term remodeling without triggering a new depolarization.

Subsequent translational and clinical studies demonstrated that CCM is associated with enhanced indices of myocardial contractility without a parallel increase in myocardial oxygen demand. CCM does not function as a pacing modality. Instead, it is conceptualized as a myocardial conditioning therapy that modulates intracellular signaling pathways involved in cardiac contraction and myocardial adaptation [15].

At the cellular and molecular levels, CCM has been shown to influence calcium handling and gene expression profiles within failing myocardium, shifting signaling pathways toward patterns more closely aligned with non-failing tissue. These effects involve regulators of excitation–contraction coupling, calcium homeostasis, and stress-response signaling [17,18]. Integrative mechanistic analyses suggest that the biological impact of CCM arises from the convergence of several adaptive processes, including improved intracellular calcium cycling, attenuation of maladaptive fetal-gene activation, and restoration of coordinated contractile signaling [18,21].

Functional imaging and metabolic studies further indicate that CCM may enhance myocardial efficiency and oxidative metabolism, providing physiologic support for observed improvements in exercise tolerance and symptom burden [20]. Taken together, these findings support the concept of CCM as a form of bioelectronic myocardial conditioning, in which repeated, precisely timed electrical inputs promote structural and functional adaptation rather than short-lived augmentation of contractile force [4,18,21].

Beyond individual mechanistic pathways, contemporary clinical syntheses emphasize that these biological effects translate into a distinct therapeutic profile, positioned between purely hemodynamic augmentation and electrical resynchronization strategies, with implications for patient selection and downstream clinical outcomes [12,22].

Although the precise molecular “sensor” responsible for transducing CCM-delivered electrical signals into sustained intracellular adaptation has not been fully elucidated, current evidence suggests that calcium-dependent signaling plays a central integrative role. Experimental and translational studies indicate that CCM alters sarcoplasmic reticulum calcium cycling and calcium transient dynamics, which may serve as an initial intracellular signal linking non-excitatory electrical input to downstream biological effects. In this context, calcium-sensitive kinases, particularly Ca2+/calmodulin-dependent protein kinase II (CaMKII), have been implicated as potential mediators connecting altered calcium handling to changes in excitation–contraction coupling and gene expression. 

Downstream signaling pathways associated with myocardial growth, survival, and metabolic efficiency may also be engaged. Preclinical and human myocardial studies have demonstrated activation of pathways such as PI3K/Akt signaling, which are known to regulate cardiomyocyte hypertrophy, cell survival, and transcriptional remodeling. These pathways may contribute to the partial normalization of maladaptive gene expression profiles observed with chronic CCM therapy. Importantly, these mechanisms are not mutually exclusive and likely represent a convergent signaling network rather than a single linear cascade, underscoring that CCM functions as a bioelectronic myocardial conditioning therapy rather than an acute inotropic stimulus.

Taken together, available mechanistic and clinical evidence support a conceptual framework in which cardiac contractility modulation functions as a form of bioelectronic myocardial conditioning rather than an acute excitatory or purely hemodynamic intervention. Subthreshold electrical stimulation delivered during the absolute refractory period appears to influence excitation–contraction coupling primarily through electrochemical modulation of intracellular calcium handling, with calcium-sensitive signaling pathways acting as key integrators and downstream adaptive remodeling processes contributing to sustained functional benefit.

## 4. Clinical Evidence

The following Table 2 shows the clinical evidence (Table 2).

### 4.1. Early Clinical Studies and Proof-of-Concept

Initial clinical investigations conducted in Europe provided foundational insights into the feasibility of cardiac contractility modulation in patients with chronic systolic heart failure. These early experiences demonstrated that repeated CCM delivery was technically feasible and generally safe. And it was associated with improvement of 6 min walk distance, peak VO_2_, or MLWHFQ scores [4,7,10,13,24].

Beyond feasibility, these studies played an important role in refining patient selection criteria and outcome measures, thereby informing the design and methodological framework of subsequent randomized investigations [4,7,10,13,24]. The clinical evidence supporting cardiac contractility modulation has been generated primarily in patients with LVEF between approximately 25% and 45%, a range that encompasses the upper spectrum of HFrEF and the lower spectrum of HFmrEF.

### 4.2. FIX-HF-5C Randomized Confirmatory Evidence

The FIX-HF-5C trial represented a key step in the clinical evaluation of CCM by prospectively assessing its effects in symptomatic heart failure patients with narrow QRS duration and moderately reduced left ventricular systolic function. In this randomized setting, CCM therapy was associated with improvements in objective measures of exercise capacity as well as patient-reported assessments of health status when compared with optimized medical therapy alone [4].

Consistent advantages were also observed across secondary domains, including functional classification and disease-specific quality-of-life instruments, supporting the clinical relevance of these findings [4]. Together, these results strengthened signals suggested in earlier exploratory analyses and contributed to subsequent refinement of responder profiles and target LVEF ranges for CCM therapy [4,7].

### 4.3. Two-Lead Systems and Contemporary Delivery (FIX-HF-5C2)

Building on earlier platform designs, the FIX-HF-5C2 study evaluated a simplified two-lead CCM system developed to reduce procedural complexity while preserving therapeutic delivery. This prospective investigation demonstrated that elimination of the atrial lead was feasible and did not compromise procedural safety or short-term clinical performance [7].

Patients treated with the two-lead configuration experienced improvements in functional status and health-related quality of life, supporting the adaptability of CCM therapy to contemporary implantation strategies and routine clinical practice settings [7].

The simplified two-lead CCM system evaluated in FIX-HF-5C2 eliminates the atrial lead and relies on ventricular sensing to time CCM signal delivery. While this design demonstrated non-inferior improvements in functional status and quality of life compared with prior systems, it introduces specific operational considerations. In the absence of atrial sensing, accurate timing of CCM delivery depends on stable ventricular rhythm and reliable sensing of intrinsic activation.

Accordingly, while the two-lead CCM system demonstrated non-inferior symptomatic benefit in FIX-HF-5C2, its performance remains dependent on rhythm stability and reliable ventricular sensing. These considerations underscore the importance of appropriate patient selection and post-implant management rather than representing an inherent limitation of the simplified lead configuration.

### 4.4. Meta-Analyses and Consolidated Outcomes

To synthesize evidence across heterogeneous study designs, an individual patient data meta-analysis pooled randomized and prospective CCM cohorts. This analysis demonstrated consistent improvements in exercise capacity and quality-of-life measures and suggested a lower burden of heart failure–related hospitalizations among patients meeting responder characteristics (Table 2) [14].

By harmonizing patient-level variables and endpoints, such pooled analyses help mitigate limitations inherent to smaller early studies and strengthen confidence in the reproducibility of symptomatic and functional benefits associated with CCM therapy [14].

### 4.5. Real-World Registries and Longer-Term Outcomes

Longer-term effectiveness of CCM has been further explored through real-world registry data, most prominently from the CCM-REG program, which examined outcomes in patient populations broadly aligned with clinical trial responder profiles. Over extended follow-up, registry participants demonstrated sustained improvements in symptoms and a reduced burden of heart failure-related hospitalizations [18,19,25].

Although the observational nature of registry analyses limits causal inference, comparative assessments suggested survival outcomes that were favorable relative to predicted risk [25]. Complementary long-term observational cohorts have similarly reported persistent gains in functional class, quality of life, and ventricular systolic performance, supporting the durability of CCM therapy in appropriately selected patients [23,26].

## 5. Patient Selection: Who Benefits Most?

### 5.1. Positioning Within HF Guidelines and Contemporary Care

In contemporary heart failure management, optimization of pharmacologic therapy remains foundational, with device-based interventions introduced selectively to address residual clinical limitations. Within this framework, cardiac contractility modulation occupies a complementary role for patients who remain symptomatic despite medical therapy but do not meet established criteria for resynchronization-based strategies [1,2,3].

Clinical trial populations and regulatory considerations have informed this positioning, generally favoring individuals with persistent symptoms, preserved intrinsic ventricular activation, and a degree of systolic dysfunction that does not routinely prompt CRT consideration [7,9,10,27]. Emerging reimbursement models that link access to prospective evidence generation may further influence how CCM is incorporated into routine practice and evaluated at a population level [28].

### 5.2. Practical Candidate Profile (Evidence-Aligned)

From a practical perspective, candidates for CCM typically include patients with ongoing functional limitation despite optimized therapy, moderate impairment of left ventricular systolic function, and electrocardiographic features that do not support resynchronization approaches. Rather than reliance on a single physiologic parameter, effective selection integrates symptom burden, functional reserve, and the anticipated ability to deliver therapy consistently [7,10,27].

In selected circumstances, CCM may also be considered for patients unable to tolerate full-dose guideline-directed medications because of hypotension or renal constraints, provided that careful clinical judgment and structured follow-up are applied [29]. The presence of atrial fibrillation does not preclude therapy but requires heightened attention to sensing reliability and programming strategy, underscoring the importance of experience in both patient selection and device management [25]. Subgroup-oriented analyses further suggest that response to CCM may vary across ejection fraction strata and rhythm status, reinforcing the need for nuanced, individualized selection [30].

**Figure 2:**
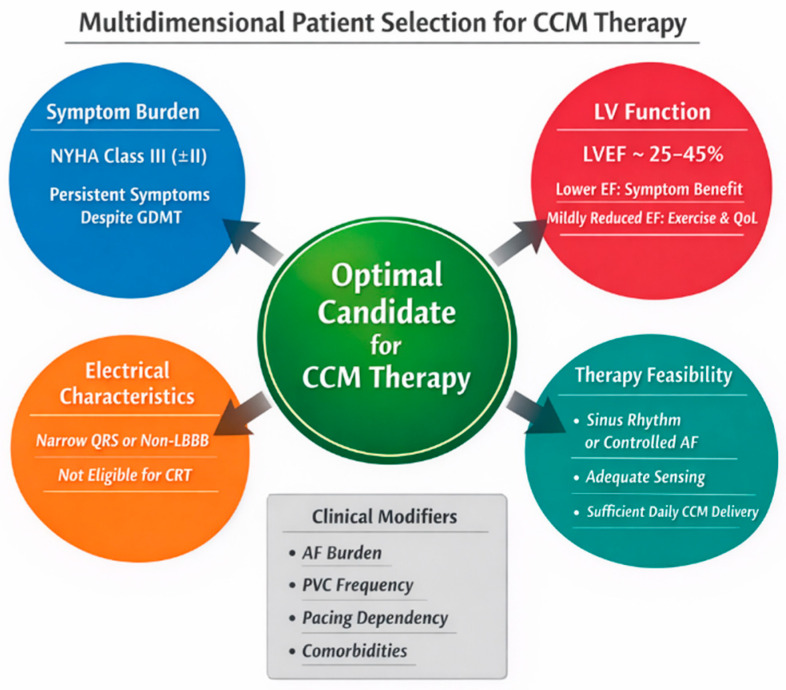
Multidimensional integration of clinical parameters for patient selection in cardiac contractility modulation (CCM) therapy (Courtesy of AI, ChatGPT 5.2). Patient selection for CCM is based on the integration of multiple clinical dimensions rather than a single parameter. Key components include persistent symptom burden (typically New York Heart Association class III despite guideline-directed medical therapy), left ventricular ejection fraction generally in the range of approximately 25–45%, electrical characteristics not qualifying for cardiac resynchronization therapy, and the feasibility of achieving adequate daily CCM therapy delivery. These factors should be considered in combination, with additional clinical modifiers such as rhythm status and comorbidity profile informing individualized decision-making. This figure is original artwork created specifically for this manuscript using AI-assisted illustration (ChatGPT 5.2, developed by OpenAI, Inc., San Francisco, CA, USA). It is not adapted from any previously published material, and no external permissions are required.

In clinical practice, patient selection for CCM requires integration of multiple dimensions rather than reliance on a single parameter. For example, an ideal candidate typically presents with persistent symptoms corresponding to New York Heart Association class III despite guideline-directed medical therapy, in combination with a left ventricular ejection fraction in the range of approximately 25–45%. Importantly, patients at the lower end of this EF spectrum may derive symptomatic benefit even in the absence of significant reverse remodeling, whereas those with mildly reduced EF often demonstrate improvements in exercise tolerance and quality of life. These considerations should be interpreted alongside rhythm status, QRS duration, and the ability to achieve adequate daily CCM therapy delivery, underscoring the need for individualized, multidimensional patient assessment (Figure 2).

The commonly cited evidence-aligned LVEF range for CCM (approximately 25–45%) reflects both pathophysiological considerations and responder enrichment observed in clinical trials. At lower LVEF values, impaired excitation–contraction coupling, altered calcium handling, and reduced contractile reserve provide a biological substrate in which CCM-mediated myocardial conditioning may exert meaningful effects. As LVEF approaches the preserved range, these abnormalities tend to be less pronounced, potentially attenuating the incremental benefit of CCM and contributing to the apparent upper boundary observed in clinical studies.

Patients with heart failure with mildly reduced ejection fraction (HFmrEF; LVEF 41–49%), therefore, represent a transitional phenotype. In this group, CCM should generally be considered only after optimization of guideline-directed medical therapy, including agents with established prognostic benefit such as sodium–glucose cotransporter 2 inhibitors, and after appropriate evaluation for reversible or disease-specific contributors to symptoms, such as myocardial ischemia or infiltrative cardiomyopathies. In carefully selected, highly symptomatic HFmrEF patients who remain limited despite these measures, CCM may be considered as a complementary option, recognizing that supporting evidence in this population remains preliminary.

From an operational standpoint, patient selection for CCM should incorporate explicit electrical, rhythm-related, and clinical criteria aligned with pivotal trials and regulatory indications. In major randomized studies, CCM was primarily evaluated in patients with narrow QRS duration, typically defined as QRS < 130 ms and most commonly <120 ms, in whom conventional CRT was not indicated. Patients with typical left bundle branch block or QRS prolongation meeting CRT criteria should generally be directed toward resynchronization-based therapies before CCM consideration.

Rhythm status is an important modifier rather than an absolute determinant of eligibility. CCM can be delivered in sinus rhythm or atrial fibrillation, provided that reliable ventricular sensing and adequate daily therapy delivery (generally >70–80% of intended CCM signals) can be achieved. In patients with atrial fibrillation, effective ventricular rate control and low burden of irregularity are essential to ensure consistent CCM delivery; uncontrolled AF with rapid or highly variable ventricular response may substantially limit therapy effectiveness.

Several clinical conditions warrant exclusion or heightened caution. These include frequent premature ventricular complexes or nonsustained ventricular tachycardia that interfere with sensing, anticipated high right ventricular pacing burden, advanced chronic kidney disease or active systemic infection increasing procedural risk, and clinical scenarios in which stable transvenous lead implantation or long-term device management is not feasible. Collectively, these operational criteria emphasize that CCM candidacy depends not only on symptom severity and ejection fraction but also on the practical feasibility of delivering consistent, high-quality therapy in routine clinical practice.

### 5.3. Exclusions and Cautions

Patients who clearly meet criteria for established resynchronization therapies—particularly those with typical left bundle branch block and marked QRS prolongation—should generally be evaluated for those modalities first, given the robust outcomes data supporting their use [2,3].

In advanced heart failure settings, including evaluation for transplantation or durable mechanical circulatory support, CCM may be considered only in carefully selected scenarios. In such cases, its role is best defined through multidisciplinary assessment, with recognition that evidence remains limited and individualized decision-making is essential [6].

## 6. Implantation and Programming: Key Practical Considerations

Effective delivery of cardiac contractility modulation requires coordinated implantation and programming strategies that ensure consistent septal signal delivery and reliable sensing. Procedural success is influenced by accurate lead positioning within the right ventricular septum and precise alignment of timing parameters with myocardial refractoriness to maximize therapy delivery while avoiding excitatory effects [4,27].

Post-implant programming and follow-up are integral to maintaining effective therapy exposure over time. Routine evaluation of sensing integrity, therapy duty cycle, and lead performance enables timely adjustment of device settings, particularly in the setting of rhythm variability, frequent ectopy, or intermittent signal interruption [4,27,31]. These operational considerations emphasize that sustained CCM benefit depends not only on implantation technique but also on structured, rhythm-aware device management.

Recent hardware developments—including reduced generator size, rechargeable power sources, and expanded programming interfaces—are designed to improve patient convenience and long-term usability. However, their clinical impact remains contingent on device generation, regional availability, and integration into local implantation and follow-up workflows [11].

In contemporary practice, CCM implantation is typically performed using a transvenous approach analogous to other right-sided cardiac implantable electronic device procedures. One or two dedicated ventricular leads are positioned along the right ventricular septum, most commonly in the mid- to basal septal region, to optimize signal delivery to the interventricular septum. Septal positioning is generally confirmed using a combination of fluoroscopic projections (right anterior oblique and left anterior oblique views), characteristic paced QRS morphology when pacing is temporarily enabled, and lead stability parameters.

Reliable sensing and consistent therapy delivery are central to CCM effectiveness. In clinical practice, low daily therapy percentage is most commonly attributable to rhythm-related factors rather than device malfunction. Frequent premature ventricular complexes, atrial fibrillation with rapid or irregular ventricular response, intermittent sensing instability, or suboptimal timing of CCM signal delivery relative to myocardial refractoriness may all reduce effective therapy exposure. Troubleshooting typically involves optimization of sensing thresholds, adjustment of timing parameters, management of ventricular ectopy or rate control, and reassessment of lead position when necessary.

Post-implant follow-up is structured to ensure early detection of sensing or delivery issues and to optimize long-term therapy exposure. An initial device interrogation is commonly performed within 2–4 weeks after implantation, followed by reassessment at 3–6 months and at regular intervals thereafter. Follow-up evaluations focus on therapy delivery percentage, rhythm stability, lead integrity, and symptom response, with programming adjustments guided by both device diagnostics and clinical status.

The procedural risk profile of CCM implantation is broadly comparable to that of other transvenous CIED procedures. Reported complications are predominantly related to lead placement or device pocket issues, including lead dislodgement, pocket hematoma, or infection, while therapy-specific proarrhythmic effects have not emerged as a dominant safety concern. When performed in experienced centers, CCM implantation demonstrates a safety profile similar to conventional pacemaker or implantable cardioverter-defibrillator procedures, reinforcing its feasibility within established device programs.

## 7. Clinical Outcomes and Endpoints

### 7.1. Symptoms and Quality of Life

Evaluation of CCM has placed particular emphasis on patient-reported outcomes, reflecting the symptom-driven nature of heart failure morbidity. In clinical studies enrolling patients most likely to respond to therapy, CCM has been associated with favorable changes in disease-specific quality-of-life measures and symptom-based functional classifications [7,24,27]. These patient-reported benefits capture dimensions of well-being and daily limitation that are not fully reflected by conventional physiologic metrics and align closely with outcomes prioritized by individuals living with chronic heart failure [1,7]. The convergence of patient-reported benefits and objectively quantified functional improvements strengthens the clinical interpretation of CCM-associated effects, linking subjective symptom relief with measurable physiologic performance.

In randomized clinical trials evaluating CCM, improvements in functional capacity and quality of life have been consistently observed across studies enrolling approximately 150–400 patients, with follow-up durations typically ranging from 6 to 12 months. Primary prespecified endpoints in these trials have included peak oxygen consumption, six-minute walk distance, New York Heart Association functional class, and disease-specific quality-of-life measures, such as the Minnesota Living with Heart Failure Questionnaire.

In contrast, observations regarding reductions in heart failure–related hospitalizations or favorable survival compared with predicted risk have largely emerged from registry-based analyses with longer follow-up periods, often extending beyond one year. These outcomes were not prespecified primary endpoints in randomized trials and should therefore be interpreted cautiously, particularly given the potential influence of confounding and selection bias inherent to non-randomized study designs.

### 7.2. Exercise Capacity and Objective Functional Performance

Objective assessments of physical performance have complemented subjective symptom evaluation in CCM studies. In selected cohorts, CCM therapy has been associated with improvements in cardiopulmonary exercise testing parameters and standardized submaximal performance measures, supporting a mechanism centered on myocardial contractile modulation rather than electrical resynchronization [7,10,27]. Such objective gains are particularly relevant for patients who remain exercise-limited despite optimized medical therapy and do not meet established criteria for resynchronization-based interventions [7,27].

### 7.3. Hospitalizations and Longer-Term Trajectory

Beyond symptomatic endpoints, analyses incorporating registry data and pooled patient-level evidence have explored the impact of CCM on clinical stability over time. These investigations suggest a potential association between CCM therapy and a lower burden of heart failure-related hospitalizations in appropriately selected populations [14,19,25]. Given the strong link between hospitalization frequency, subsequent mortality risk, and health-care utilization, such findings are of particular interest. Nevertheless, interpretation remains tempered by the relative scarcity of large-scale randomized trials powered for hard outcomes, underscoring the need for continued prospective evaluation [1,14].

Importantly, the current evidence base supporting cardiac contractility modulation differs across outcome domains. Randomized clinical trials of CCM have been primarily designed and powered to assess functional capacity and health-related quality-of-life endpoints, such as peak oxygen consumption and disease-specific symptom scores. In contrast, signals suggesting reductions in heart failure-related hospitalizations or favorable survival relative to predicted risk have largely emerged from observational studies and prospective registries.

While these real-world findings are clinically encouraging, they remain subject to inherent limitations, including residual confounding, selection bias, and regression to the mean. As such, associations between CCM therapy and “hard” clinical outcomes should be interpreted as hypothesis-generating rather than definitive evidence of causal benefit. Future randomized studies specifically powered for hospitalization and mortality endpoints will be required to more precisely define the impact of CCM on long-term clinical outcomes.

The absence of a large randomized trial powered for hard clinical endpoints represents a recognized limitation in the evidence base supporting cardiac contractility modulation. However, the potential for clinical adoption of CCM should be interpreted within the broader context of contemporary heart failure therapeutics. In selected patient populations with persistent symptoms despite guideline-directed medical therapy and limited device-based alternatives, reproducible improvements in exercise capacity and health-related quality of life may constitute a clinically meaningful benefit, even in the absence of definitive mortality data.

Designing and executing a large outcome-driven randomized trial for CCM poses several challenges. These include heterogeneity of the target population, high background use of contemporary pharmacologic therapies that attenuate incremental event rates, and the logistical and ethical complexity of implementing sham-controlled device trials. In addition, the symptomatic focus of CCM, variability in therapy delivery due to rhythm-related factors, and the large sample size and prolonged follow-up required to detect differences in hospitalization or survival all represent substantial practical barriers. As a result, current observational and registry data should be viewed as hypothesis-generating, while future studies may require innovative trial designs or pragmatic approaches to more fully define the long-term outcome impact of CCM.

## 8. Safety, Procedural Considerations, and Arrhythmia-Related Issues

Implantation of cardiac contractility modulation systems involves procedural considerations similar to those of other transvenous cardiac implantable devices, underscoring the importance of meticulous technique, operator experience, and structured post-implant surveillance to mitigate lead- and pocket-related complications [7,23,27]. Reported adverse events have been largely procedural in nature, with safety profiles comparable to established device therapies when CCM is implemented within experienced programs.

Although CCM signals are delivered exclusively during the myocardial refractory period and are not intended to induce depolarization, effective therapy delivery may be influenced by rhythm-related factors. Variability in atrial rhythm, frequent ventricular ectopy, or sensing instability can affect the consistency of CCM delivery and often necessitate individualized programming strategies and closer follow-up rather than reflecting proarrhythmic risk [4,25].

Across early clinical investigations and contemporary cohorts, therapy-specific arrhythmic adverse effects have not emerged as a dominant safety concern. Instead, overall safety outcomes appear primarily driven by procedural and device-related factors, supporting the feasibility of CCM therapy when integrated into structured device workflows with appropriate rhythm-aware management [7,23,27].

## 9. Emerging Directions: CCM-D, Broader Populations, and Health-System Adoption

### 9.1. CCM in HFmrEF/HFpEF and Other Emerging Niches

Interest in extending cardiac contractility modulation beyond the conventional HFrEF responder population has increased, particularly in patients with heart failure with mildly reduced or preserved ejection fraction. Although CCM was developed and validated primarily in reduced ejection fraction cohorts, its non-excitatory myocardial conditioning mechanism provides a biologically plausible rationale for benefit across a broader spectrum of myocardial dysfunction. However, translation into clinical practice will require robust evidence identifying phenotypes most likely to derive meaningful functional improvement. Contemporary perspectives emphasize multidimensional selection strategies—integrating symptom burden, functional capacity, rehabilitation response, and physiologic profiling—to refine targeting as potential indications continue to evolve [18,21,32,33].

Extension of cardiac contractility modulation into heart failure with preserved ejection fraction should be approached with careful phenotypic selection, given the heterogeneity of HFpEF and its predominant association with diastolic dysfunction and abnormal ventricular–vascular coupling. Rather than targeting HFpEF as a uniform entity, the most mechanistically plausible candidates for CCM are likely to be patients with evidence of latent or dynamic systolic impairment, reduced contractile reserve, or abnormal myocardial energetics despite preserved resting LVEF.

Such phenotypes may include patients who demonstrate exercise-induced systolic dysfunction, impaired longitudinal strain, blunted augmentation of stroke volume during stress, or disproportionate exercise intolerance relative to resting echocardiographic measures. In these subgroups, CCM-mediated myocardial conditioning may theoretically enhance excitation–contraction coupling efficiency and energetic utilization, thereby improving functional capacity. Importantly, these concepts remain exploratory, and the role of CCM in HFpEF should currently be viewed as hypothesis-generating, warranting evaluation only within rigorously designed, phenotype-enriched clinical trials.

### 9.2. Integrated CCM–Defibrillator (CCM-D) Systems: Technological Evolution and Early Clinical Experience

As the scope of patients considered for CCM expands, device strategies capable of accommodating increasing clinical complexity have gained importance. Integrated CCM–defibrillator (CCM-D) systems represent a technological response to this need, combining symptomatic heart failure support with protection against malignant ventricular arrhythmias within a single platform. By consolidating these functions, CCM-D systems are conceptually designed to reduce procedural burden, avoid parallel device implantation, and streamline long-term device management. From a clinical standpoint, this approach is particularly relevant for ICD-indicated patients who remain functionally limited despite optimized medical therapy, addressing an unmet need in contemporary heart failure care and providing a foundation for broader integration of CCM into advanced device-based treatment pathways [6,34,35,36].

Integration of cardiac contractility modulation and defibrillation within a single CCM-D platform raises theoretical concerns regarding interaction between high-voltage CCM pulses and implantable cardioverter–defibrillator sensing algorithms. Although CCM signals are of relatively high amplitude, they are delivered during the absolute refractory period and are temporally separated from ventricular arrhythmia sensing windows, which substantially reduces the likelihood of inappropriate arrhythmia detection.

To date, available clinical experience with integrated CCM-D systems has not demonstrated a dominant signal of clinically relevant over-sensing or under-sensing of ventricular arrhythmias when devices are appropriately programmed. Key programming considerations unique to CCM-D devices include careful selection of sensing vectors, optimization of blanking and refractory periods, and post-implant verification of arrhythmia detection integrity under active CCM delivery. In addition, routine follow-up interrogation is essential to confirm stable sensing performance, particularly in patients with frequent ventricular ectopy or changing rhythm characteristics. These measures collectively support safe coexistence of CCM delivery and defibrillation within a unified device platform.

### 9.3. Coverage and Implementation Landscape

The adoption of CCM will be influenced not only by clinical efficacy but also by health-system factors such as reimbursement structures, procedural logistics, and value assessment. Coverage models linking reimbursement to prospective evidence generation highlight the importance of high-quality real-world data on outcomes, utilization, and cost-effectiveness. Parallel health-technology assessments continue to inform system-level perspectives on appropriate patient selection and sustainable implementation [28,36,37].

### 9.4. CCM-D (INTEGRA-D): Early Performance and Clinical Signal

Beyond conceptual rationale, early data from the INTEGRA-D program provide an initial clinical signal supporting the feasibility of delivering CCM within an integrated defibrillator platform. These reports emphasize successful coexistence of CCM delivery with sensing and defibrillation requirements, while suggesting preservation or improvement of functional status after implantation in ICD-eligible HFrEF patients. Although these observations remain preliminary, they position CCM-D as a practical extension of CCM therapy, pending prospective confirmation of durability, safety, and net clinical benefit [6].

In patients with an existing implantable cardioverter-defibrillator who develop progressive heart failure symptoms and meet criteria for cardiac contractility modulation, optimal management requires close collaboration between heart failure cardiologists and electrophysiologists. The decision to pursue an integrated CCM–defibrillator (CCM-D) system versus implantation of a standalone CCM device should be individualized and informed by several key considerations.

Upgrading to a CCM-D system may be favored when ICD generator replacement is already anticipated, venous access is limited, or minimizing additional leads and device pockets is desirable. In contrast, adding a standalone CCM device may be reasonable when the existing ICD system is relatively new, arrhythmic risk is stable, and procedural risk associated with device extraction or replacement is deemed high. Additional factors influencing this decision include the patient’s burden of ventricular arrhythmia, likelihood of future ICD therapies, anatomical feasibility, and patient preferences regarding procedural complexity.

Importantly, neither approach should be viewed as universally superior. Rather, effective integration of CCM into the care of ICD-treated patients depends on multidisciplinary evaluation that balances heart failure symptom burden, arrhythmic risk, and technical considerations, underscoring the complementary roles of heart failure and electrophysiology expertise in contemporary device-based management.

## 10. Health System Considerations and Future Challenges

From a healthcare system perspective, the cost-effectiveness of cardiac contractility modulation is influenced by its relatively high upfront device and implantation costs, balanced against the durability of symptomatic benefit and potential downstream reductions in heart failure–related resource utilization. Clinical trials and registries suggest that improvements in quality of life and exercise capacity typically emerge within the first few months after implantation, implying a relatively short time to symptomatic benefit. However, economic justification at the system level would require that these benefits be sustained and accompanied by a meaningful reduction in recurrent heart failure hospitalizations over a longer time horizon.

Although definitive cost-effectiveness analyses for CCM remain limited, modeling frameworks suggest that avoidance of even a modest number of heart failure hospitalizations over several years could substantially offset initial device costs. The introduction of rechargeable CCM generators has the potential to further improve this balance by extending device longevity and reducing the frequency of generator replacement procedures, thereby lowering cumulative procedural costs and associated risks. Importantly, the economic value of CCM is likely to be highly sensitive to patient selection, with the greatest cost-effectiveness expected in patients who experience frequent hospitalizations or high symptom burden prior to therapy. As such, robust prospective health economic analyses remain an important unmet need.

## 11. Limitations

This review has several limitations. First, the strongest evidence supporting CCM relates to functional capacity and quality-of-life outcomes, whereas large randomized trials powered for hard clinical endpoints remain limited. Second, much of the long-term outcome data derive from observational registries, which are subject to residual confounding. Finally, CCM technology and patient selection strategies continue to evolve, and future studies may further refine its role in heart failure management.

## 12. Conclusions

Cardiac contractility modulation (CCM) represents a bioelectronic therapeutic option for patients with symptomatic heart failure who do not meet criteria for cardiac resynchronization therapy. Across randomized clinical trials, the most robust and consistent evidence supporting CCM relates to improvements in exercise capacity and health-related quality of life, outcomes that directly reflect patient-centered benefit.

In contrast, evidence suggesting reductions in heart failure–related hospitalizations or favorable effects on survival is less definitive and has largely emerged from observational studies and prospective registries rather than trials specifically powered for hard clinical endpoints. As such, these findings should be interpreted cautiously and viewed as supportive but not conclusive.

Emerging developments—including integrated CCM–defibrillator platforms and exploratory application of CCM in patients with mildly reduced or preserved ejection fraction—represent promising but still preliminary directions. At present, the strongest and most reproducible evidence supporting CCM lies in patient-centered outcomes such as exercise capacity and quality of life, whereas its impact on hospitalization and survival remains less definitively established. Future prospective studies, ideally incorporating rigorous phenotypic enrichment and appropriate clinical endpoints, will be required to further define the long-term role of CCM across the evolving spectrum of heart failure care.

## Figures and Tables

**Table 1:** Proposed biological and physiological mechanisms underlying the effects of cardiac contractility modulation therapy in chronic heart failure.

	Mechanism	Key Effects	Supporting Evidence
Electrical † [8,12]	Non-excitatory electrical signals delivered during the absolute refractory period	Avoids triggered depolarization while influencing intracellular signaling	Preclinical canine models; human electrophysiologic studies
Cellular † [5,13,14]	Modulation of calcium handling proteins (SERCA2a, phospholamban)	Enhanced excitation–contraction coupling and contractile efficiency	Myocardial biopsy and translational studies
Molecular † [14,15,16]	Partial normalization of maladaptive gene expression (“fetal gene program”)	Reverse remodeling–like effects at the transcriptional level	Human myocardial gene-expression analyses
Metabolic † [5,17]	Improved myocardial oxidative metabolism and efficiency	Increased contractile work without excess oxygen consumption	PET and metabolic imaging studies
Structural † [13,18,19]	Favorable effects on LV remodeling	Improvement in LV function and geometry over time	Long-term clinical and registry data
Functional † [4,7,20]	Increased systolic performance without increased arrhythmogenic risk	Improved exercise capacity and symptoms	Randomized clinical trials (FIX-HF-5C, FIX-HF-5C2)

Cardiac contractility modulation (CCM) therapy delivers non-excitatory electrical impulses during the absolute refractory period, producing multi-level biological effects ranging from acute cellular calcium handling to longer-term molecular and structural remodeling. † Reference numbers [4,5,7,8,12,13,14,15,16,17,18,19,20] indicate representative studies supporting each mechanistic category. Abbreviations: CaMKII, Ca2+/calmodulin-dependent protein kinase II; PI3K/Akt, phosphoinositide 3-kinase/protein kinase B. Although a single upstream molecular “sensor” for cardiac contractility modulation (CCM) has not been definitively identified, available experimental and translational evidence suggests that alterations in intracellular calcium handling may act as a proximal integrative signal. Calcium-sensitive pathways, including CaMKII-related signaling, are implicated in linking CCM-delivered electrical inputs to changes in excitation–contraction coupling and gene expression, while downstream engagement of PI3K/Akt signaling may contribute to myocardial survival, metabolic efficiency, and transcriptional remodeling observed with chronic CCM therapy.

**Table 2:** Summary of pivotal randomized trials and real-world studies evaluating cardiac contractility modulation therapy in heart failure.

Study	Design	Patient Population, *n* (number)	Inclusion Criteria / Follow-Up	Primary Endpoint / Key Outcomes
FIX-HF-5 ‡ [10]	Randomized controlled trial	HFrEF patients (*n* = 428)	NYHA III/IV, narrow QRS / Primary assessment at 24 weeks; extended follow-up reported to 50 weeks	Initial efficacy signals in exercise capacity and QOL / Responder analysis of change in ventilatory anaerobic threshold (VAT); safety endpoint device-related AEs
FIX-HF-5C ‡ [4]	Randomized confirmatory trial	HFrEF/HFmrEF (*n* = 160; 74 CCM vs. 86 control)	NYHA III, LVEF 25–45%, non-CRT candidates / 24 weeks (assessments at 12 and 24 weeks)	Significant improvement in peak VO_2_ 6MWD, and MLWHFQ from baseline to 24 weeks / Improves exercise tolerance and quality of life in the specified group of HF patients, and leads to fewer HF hospitalizations
FIX-HF-5C2 (Optimizer Smart 2-lead) ‡ [7]	Prospective single-arm; compared vs. prior RCT controls	Symptomatic HF (*n* = 60)	FIX-HF-5C2 (2-lead system) subjects / 24 weeks	estimated difference in the change of peak VO2 from baseline to 24 weeks between FIX-HF-5C2 (2-lead system) subjects relative to control / Improved functional status and QOL; simplified implantation
CCM-REG ‡ [18]	Prospective multicenter registry	Real-world CCM recipients (*n* = 140)	FDA-aligned indication / 2 years (hospitalizations, QoL/NYHA); survival compared vs. SHFM through 3 years	Cardiovascular and HF hospitalizations, Minnesota Living with Heart Failure Questionnaire (MLHFQ) and NYHA class / Reduced HF hospitalizations; sustained symptom improvement
Long-term observational cohorts ‡ [23]	Observational registry	Advanced chronic HF (*n* = 106)	NYHA class, MLWHFQ score, 6 min walk distance, LVEF, and peak VO2 at baseline and 6 month intervals / 24 weeks Reported follow-up up to ~24 months	Durable improvements in NYHA class and LV function / safe and effective long-term symptomatic and functional improvement in heart failure

Randomized clinical trials and prospective registries consistently demonstrate that CCM therapy improves functional capacity and quality of life in carefully selected heart failure patients who remain symptomatic despite guideline-directed medical therapy and are not candidates for CRT. Real-world data further support durability of benefit and suggest potential reductions in heart failure hospitalization, while emerging CCM-D platforms may expand applicability to patients with concomitant ICD indications. ‡ Reference numbers [4,7,10,18,23] indicate the primary publications reporting the design and outcomes of each randomized trial, registry, or observational cohort.

## Data Availability

No new data were created or analyzed in this study.
